# Role of Pseudogenes in Tumorigenesis

**DOI:** 10.3390/cancers10080256

**Published:** 2018-08-01

**Authors:** Xinling Hu, Liu Yang, Yin-Yuan Mo

**Affiliations:** 1 Department of Dermatology and Institute of Translation Medicine, Affiliated the First People’s Hospital of Chenzhou of University of South China, Chenzhou 423000, China; xhu@umc.edu; 2 Cancer Institute, University of Mississippi Medical Center, Jackson, MS 39216, USA; 3 Department of Science & Research, Zhejiang Provincial People’s Hospital, Hangzhou 310014, China; yangliuqq2003@163.com; 4 Department of Pharmacology/Toxicology, University of Mississippi Medical Center, Jackson, MS 39216, USA

**Keywords:** LncRNA, pseudogene, gene regulation, ceRNA, microRNA

## Abstract

Functional genomics has provided evidence that the human genome transcribes a large number of non-coding genes in addition to protein-coding genes, including microRNAs and long non-coding RNAs (lncRNAs). Among the group of lncRNAs are pseudogenes that have not been paid attention in the past, compared to other members of lncRNAs. However, increasing evidence points the important role of pseudogenes in diverse cellular functions, and dysregulation of pseudogenes are often associated with various human diseases including cancer. Like other types of lncRNAs, pseudogenes can also function as master regulators for gene expression and thus, they can play a critical role in various aspects of tumorigenesis. In this review we discuss the latest developments in pseudogene research, focusing on how pseudogenes impact tumorigenesis through different gene regulation mechanisms. Given the high sequence homology with the corresponding parent genes, we also discuss challenges for pseudogene research.

## 1. Introduction

Accumulating evidence indicates that regulation of gene expression is much more complicated than previously anticipated. For instance, the recent discovery of large numbers of non-coding RNAs reveals that these molecules can be involved in different levels of regulation of gene expression [1,2,3,4,5]. Long non-coding RNAs (lncRNAs) are a large and diverse class of RNAs with >200 nucleotides in length and they generally lack the coding capacity. Recent studies suggest that lncRNAs are essential transcriptional and post-transcriptional regulators such that they regulate expression of protein-coding genes as well as non-coding genes. Dysregulation of lncRNAs has been linked with a number of human diseases, especially cancer [6,7].

Overwhelming numbers of lncRNAs have been reported to date [8]. LncRNAs consist of diverse groups of non-coding RNAs, such as transcribed ultraconserved regions (T-UCR) [9]; and natural antisense transcripts (NATs) that are transcribed on the opposite strand from a protein-coding gene and frequently overlap with the corresponding gene [10,11,12,13,14]; and enhancer RNAs (eRNAs) [15]. In addition, there is another important group of lncRNAs, i.e., long intergenic non-coding RNAs (lincRNAs), which are characterized by their transcriptions separated by long stretches of intergenic space [16]. Finally, a special group of lncRNAs is pseudogenes, which is the focus of this review.

Pseudogenes are evolutionally conserved, and are present in diverse organisms [17]. Compared to other members of lncRNAs, pseudogenes have been recently caught attention probably due to the finding that *PTEN* pseudogene 1 (*PTEN*P1) harbors microRNA response elements (MREs) shared by its corresponding protein-coding gene, *PTEN* [18]. Indeed, the similar phenomenon has been demonstrated in a large number of cases for pseudogenes and corresponding protein-coding genes [19,20]. In this way, pseudogenes and coding genes can talk with each other by competing for the same microRNAs, acting as competitive endogenous RNAs (ceRNAs) [21,22]. These studies highlight the significance of pseudogenes in gene regulation, which may ultimately impact various aspects of tumorigenesis. Furthermore, ceRNA is not the only mechanism by which pseudogenes can function in regulation of gene expression, as we will discuss below.

To date, a larger number of human pseudogenes have been identified. Based on updated HUGO gene nomenclature committee (HGNC) statistics (https://www.genenames.org/cgi-bin/statistics), there are over 13,000 annotated pseudogenes, although the actual number of pseudogenes could be larger than this number [21]. As detection technology advances, the number of pseudogenes will keep arising.

Although it is not clear whether all of these identified pseudogenes are expressed, at least a number of pseudogenes have been experimentally confirmed, particularly for those whose parent genes are abundantly expressed [23,24], including those with their parent genes coding for human ribosomal proteins or those involved in the glycolytic pathway [24,25]. An interesting finding of these studies is that parent gene levels affect pseudogene expression [24]. A systematic characterization by a computational pipeline analysis of transcribed pseudogenes from RNA-Seq data revealed that ~3000 pseudogenes produce non-coding RNAs under normal physiological conditions [26]. We interrogated 13,000 pseudogenes against the Cancer Genome Atlas (TCGA) dataset and found that a large number of pseudogenes are dysregulated in various types of cancer (see below), suggesting their potential role in cancer.

## 2. Types of Pseudogenes

**Figure 1:**
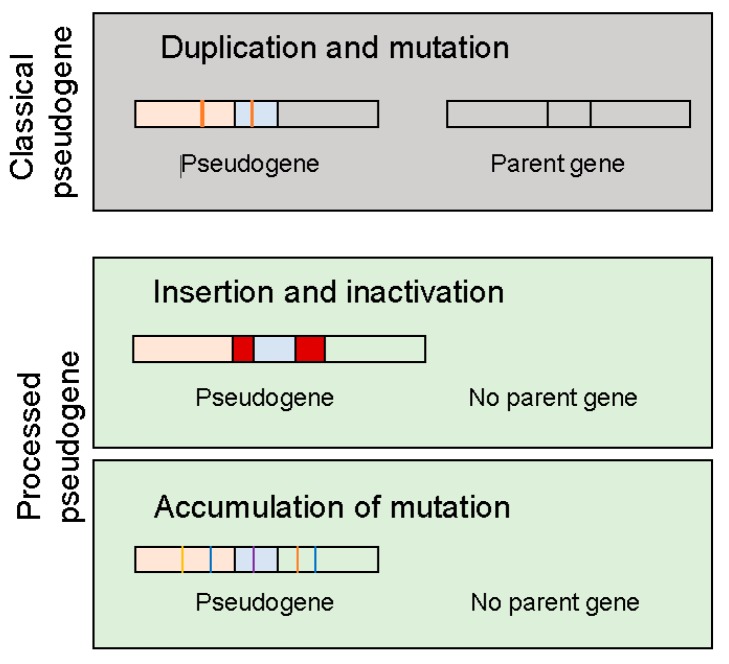
Types of pseudogenes. See detailed explanation in the text.

No matter how pseudogenes are derived from, they have lost their capability to synthesize proteins (polypeptides) due to events such as premature stop codons, splicing errors, frameshift-causing deletions and insertions. There are three types of events that could lead to the creation of pseudogenes (Figure 1): (1) duplication and mutation; (2) processing that may involve retrotransposon insertion and inactivate the coding ability and (3) accumulation of mutations such that the original gene has lost his coding capacity. In the last two cases, these unitary pseudogenes often lack functioning counterparts [21], although they may constitute only a small fraction of annotated pseudogenes in the human genome. Depending on the genomic location and how they are transcribed, pseudogenes can be processed into short interfering RNAs that regulate coding genes through the RNAi pathway or they may be able to interact with the promoter of parent genes or they may act as microRNA decoys to regulate the parent gene.

It should be pointed out that although the pseudogenes we discussed above are related to protein-coding genes, we would expect that pseudogenes can also be derived from non-coding parent genes by the similar mechanism.

## 3. Functional Mechanism of Pseudogenes

Based on our current understanding, pseudogenes can regulate gene (not necessarily parent gene) expression at transcriptional and post-transcriptional level. At the transcriptional level, pseudogene may interact with a gene promoter. For example, antisense RNA generated from pseudogenes can combine with sense-stranded mRNA from a homologous parent gene and either inhibit translation or lead to the formation of siRNAs that can inhibit expression of the parent gene. Post-transcriptional regulation by pseudogene is represented by their function as microRNA decoys, also known as competing endogenous RNA (ceRNA). Finally, RNA from parent genes and their homologous pseudogenes can compete for RNA binding proteins (RBPs) that may have a positive or negative effect on parent gene mRNAs, depending on the functional nature of RBPs. When the levels of pseudogene transcripts are changed, this would, in turn, lead to alterations of the parent gene mRNA levels. Therefore, pseudogenes may function as positive or negative regulators of gene expression.

### 3.1. Pseudogenes as Positive Gene Regulators

**Figure 2:**
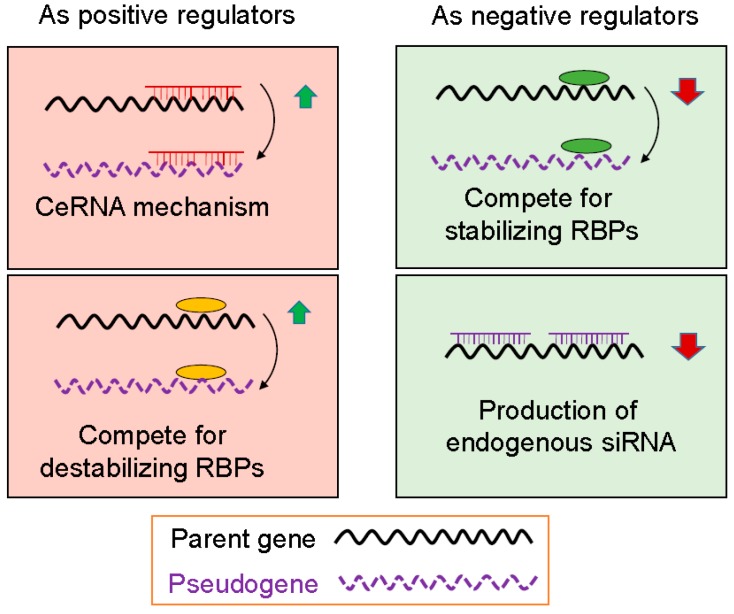
Possible mechanisms of pseudogene-mediated gene regulation. As positive regulators, pseudogenes may compete microRNA response elements (MREs) with the parent genes for the same microRNAs or same destabilizing RNA binding proteins (RBPs). As a result, levels of the parent genes are increased. As a negative regulators, pseudogenes may compete the same stabilizing RBPs with the parent genes, leading to downregulation of parent genes. Alternatively, pseudogenes may transcribe into endogenous siRNAs that can bind to any region of the parent genes, and suppress expression of the parent genes.

In this scenario, there is a positive correlation between pseudogene and its parent gene. A well-characterized mechanism responsible for this type of action is gene regulation by ceRNA through which the pseudogene transcripts share same MREs and compete with the parent transcripts for same microRNAs (Figure 2). Overwhelming evidence indicates that this is a new layer of post-transcriptional regulation occurred in a variety of organisms [27,28,29]. In this regulatory system, multiple RNA transcripts from pseudogenes and parental genes may contain shared MREs for common microRNAs and thus, these transcripts could co-regulate one another. Our own studies also indicate that a number of lncRNAs can participate in ceRNA regulatory network [30,31].

*PTEN*P1 is the first example of pseudogenes that can regulate its parent gene *PTEN* through ceRNA mechanism [18]. In this regard, *PTEN*P1 functions as decoys to adsorb microRNAs targeting the *PTEN* tumor suppressor for degradation. For example, there are perfectly conserved seed matches for the *PTEN* targeting microRNAs such as miR-17, miR-21, miR-214, miR-19 and miR-26 families. Thus, like *PTEN*, *PTEN*P1 also functions as a tumor suppressor, and *PTEN*P1 upregulation causes growth inhibition of tumor cells. 

As a matter of fact, ceRNA is a predominant mechanism reported for a large number of lncRNAs including pseudogenes. However, it should be pointed out that since large body of these studies are based on ectopic expression or transgenic expression of lncRNA/pseudogene, it remains to be determined whether this ceRNA network functions efficiently under physiological conditions. The abundance of a given microRNA in the cell may be key for ceRNA mechanism. For example, low expressed microRNAs can be susceptible to ceRNAs; for highly expressed microRNAs it might be difficult to achieve such regulation. Large changes in target abundances may diminish the ability of individual transcripts to disrupt the activity of highly expressed microRNAs [28]. In addition, protein-coding genes and lncRNAs can serve as microRNA host genes [32]. This may also apply to pseudogenes. For example, miR-220 and miR-492 were identified to be within a processed pseudogene [33]. The implication of this finding is that these embedded microRNAs, like other types of microRNAs, may be involved in ceRNA network.

RBPs play an important role in gene expression at the post-transcriptional level. For instance, many mRNA species carry AU-rich elements (AREs) at the 3′ untranslated region (3′-UTR). AREs are one of the most common determinants of RNA stability in mammalian cells by various RBPs including stabilizing and destabilizing factors. As stabilizing factors such as HuR, they interact with AREs to stabilize the mRNA whereas destabilizing factors, such as AUF1 and TTP, bind to AREs to destabilize the mRNA (Figure 2). Given the ability of pseudogene RNAs to interact with RBPs, it is anticipated that this type of interaction between pseudogenes and RBPs will impact the amount of RBPs in the pool and ultimately impact the function of those RNAs that share the same binding sites with those of pseudogenes. In this way, the mRNA molecules are either stabilized or destabilized. For pseudogenes to function as positive regulators, involved RBPs are those that can destabilize target mRNAs so that the stability of the target mRNA is increased when their RBPs are bound to pseudogenes (Figure 2).

It is known that the mRNA level for many genes is regulated by stabilizing/destabilizing RBPs in the 3′-UTR [34]. Although there is little information available regarding the interaction of pseudogenes with RBPs to positively regulate target gene expression, a relevant example is the interaction of Linc-RoR and Myc mRNA to compete for AUF1 destabilizing factor. Myc mRNA stability is controlled by several RBPs [35] and this regulation is critical to Myc-mediated tumorigenesis because the half-life of Myc mRNA in cancer cells is significantly longer than in normal cells [36]. Our recent study suggests that Linc-RoR interacts with heterogeneous nuclear ribonucleoprotein (hnRNP) I (stabilizing factor) and AUF1 (destabilizing factor), respectively, with an opposite consequence to their interaction with c-Myc mRNA [37]. In particular, interaction of Linc-RoR with AUF1 inhibits AUF1 to bind to c-Myc mRNA [37]. Thus, we anticipate that many pseudogene and parent gene pairs could also be subject to this type of competition that may or may not involve additional factors including lncRNAs.

Other mechanisms of pseudogene-mediated gene regulation may involve epigenetic regulation or protein translation. Of particular interest, *PTEN*P1 can also play a role in epigenetic regulation of *PTEN*. It turns out that transcription of *PTEN*P1 produces sense and antisense transcripts that exhibit transcriptional and post-transcriptional modulation of *PTEN* expression, respectively. It is well documented that *PTEN*P1 sense transcript acts as a decoy for *PTEN* targeting microRNAs; on the other hand, *PTEN*P1 can make two anti-sense transcripts, i.e., α and β isoforms, which have different functions [38].

The α isoform negatively regulates *PTEN* transcription and the β isoform positively regulates *PTEN* mRNA post-transcriptionally through *PTEN*P1 sense. For example, knockdown of the α isoform causes upregulation of *PTEN*, while its overexpression suppresses *PTEN* mRNA levels. Mechanistically, the α isoform functions in trans, localizing to the *PTEN* promoter and inhibiting *PTEN* expression. This *PTEN*P1 asRNAα (antisense RNA)-mediated repression of *PTEN* involves EZH2 and DNMT3A because it interacts with DNMT3A which is required for the deposition of repressive H3K27me3 chromatin marks at the *PTEN* promoter [39,40]. On the other hand, the β isoform can interact with *PTEN*P1 through an RNA-RNA pairing interaction, which affects *PTEN* protein output through changes of *PTEN*P1 stability and microRNA sponge activity. Disruption of this asRNA-regulated network induces cell-cycle arrest and sensitizes cells to doxorubicin [39]. These studies provide excellent examples of the complex regulation system controlling *PTEN* expression, ultimately impacting PI3K/AKT signaling.

The Oct4 gene (POU5F1) is known for its role in pluripotency and it has several splice variants as well as related pseudogenes [41]. Several of the pseudogenes are expressed in tumor specimens and cancer cell lines [42]. Among them is POU5F1B (POU domain class 5 transcription factor 1B), a processed pseudogene that is highly homologous to OCT4. Overexpression of POU5F1B in gastric cancer cells promotes colony formation in vitro and tumor growth in vivo [43]. Of interest, MYC overexpression enhances POU5F1B-induced tumorigenesis. Although there is a report that POU5F1B expression is positively correlated with the parent gene POU5F1 in prostate cancer [44], POU5F1B promotes angiogenesis and cell proliferation and inhibits apoptosis in gastric cancer [43], suggesting that POU5F1B-induced tumorigenesis may not be through regulation of its parent gene, instead directly or indirectly regulate genes related to angiogenesis or cell proliferation.

### 3.2. Pseudogenes as Negative Gene Regulators

As discussed above, stabilizing RBPs such as HuR bind to AREs to stabilize the mRNA. Since pseudogenes can compete with the parent genes for stabilizing RBPs, we would expect that the parent gene mRNA will likely be less stable such that the mRNA level decreases. For this type of competition, pseudogenes can function as negative gene regulators (Figure 2).

There are at least two examples that pseudogenes may interact with RBPs to reduce the mRNA stability of parent genes. One notable example is high mobility group A1 (HMGA1), an important nuclear factor that activates gene transcription by binding to AT-rich sequences in the promoter region of DNA. HMGA1 is abundantly expressed in all human neoplastic tissues, which is associated with poor prognosis in diverse tumors. Several studies showed that overexpression of HMGA1 drives neoplastic transformation in cultured cells, whereas suppression of HMGA1 expression inhibits oncogenic and cancer stem cell properties [45,46].

There are eight processed HMGA1 pseudogenes (HMGA1Ps) [46]. An early study showed that HMGA1 protein regulates the insulin receptor gene through a RNA binding protein αCP1, also called Poly (RC) Binding Protein 1 (PCBP1) [47,48]. Of interest, suppression of HMGA1P mRNA results in a reciprocal increase in HMGA1 mRNA stability and expression levels with a parallel correction in cell-surface insulin receptor expression and insulin binding [49]. A possible mechanism may involve αCP1, which functions as a single-stranded nucleic acid binding protein that binds preferentially to oligo dC. This protein was initially identified in the complex associated with the 3′-UTR of erythropoietin messenger RNA [50], suggesting a possible role in regulation of mRNA stability. In support of this notion, 3′-UTR of both HMGA1 and its pseudogene contains potentially important C-rich stretches [50]. Thus, it would be interesting to determine whether such a type of interaction and competition also occurs in cancer.

Another example is myosin light chain kinase pseudogene (MYLKP1) and smooth muscle myosin light chain kinase (smMLCK). In this system, MYLKP1 and smMLCK can reciprocally repress each other. MYLKP1 is highly expressed in lung adenocarcinoma cells whereas smMLCK is highly expressed in normal bronchial epithelial cells. Furthermore, MYLKP1 overexpression inhibits smMLCK expression in cancer cells by decreasing RNA stability, leading to increased cell proliferation [51]. It is possible that a RBP is involved in this competition, but it remains to be determined which specific RBP plays a role in this aspect. Given the negative correlation of expression between these two genes, it is likely that a stabilizing RBP is involved in this system.

We already mentioned that pseudogene may suppress gene expression through RNA inference (RNAi) pathway. In this regard, pseudogenes can be transcribed into antisense RNAs, some of which may function as endogenous siRNAs. For instance, bioinformatics analysis suggests that many human pseudogenes can produce small RNAs and some of which are derived from antisense strands. These small RNAs may function as siRNAs to suppress the parent gene expression [26]. Tam et al. provided further evidence that a number of endogenous siRNAs can be derived from pseudogenes in mouse [52], suggesting that pseudogenes can regulate gene expression by means of the RNAi pathway. In human hepatocellular carcinoma, pseudogene ψPPM1K is capable of transcribing two specific endogenous siRNAs, and moreover, they function as tumor suppressor by targeting other genes such as NEK8, related to the parent PPM1K [53], suggesting that pseudogenes may function independent of their parental genes. In addition to mammalian cells, a cluster of siRNAs derived from pseudogenes has been identified in African *Trypanosoma brucei* and these pseudogene-derived siRNAs can also suppress gene expression through RNAi [54]. Together, these studies suggest that endogenous siRNAs may originate from pseudogenes and regulate gene expression, further supporting the functional roles of pseudogenes in gene regulation.

Finally, pseudogenes may exert to suppress protein translation of the parent gene mRNA. An example is neuronal NOS (nNOS) pseudogene. The pseudo-NOS transcript is expressed in the central nerve system (CNS) of the snail Lymnaea stagnalis and it carries a region of significant antisense homology to neuronal NOS (nNOS), a protein-encoding mRNA. This antisense region of the pseudo-NOS prevents the translation of nNOS protein from the nNOS-encoding mRNA [55]. Unfortunately, there is no follow up as regard to how pseudo-NOS suppresses the translation of nNOS. However, it was speculated that this translation suppression could be due to the formation of stable RNA-RNA duplex molecules between the two transcripts.

## 4. Role of Pseudogenes in Cancer

Since pseudogenes can have a broad and multifaceted activity on gene expression, they are expected to play a role in human cancer. As matter of fact, most of pseudogene studies have been carried out in cancer. In particular, pseudogenes are aberrantly expressed in a variety of cancer types. In this section, we will first list a few of relatively well studied pseudogenes and discuss how they impact tumorigenesis. We will then move on expression of pseudogenes in cancer through the TCGA dataset to provide their potential clinical relevance.

Like other lncRNAs, pseudogenes were initially thought to be non-functional. However, increasing evidence indicates that they can play critical roles at multiple levels in diverse physiological and pathological processes, including parent gene-dependent or parent gene-independent regulation. Apparently, given the role of pseudogenes in regulation of parent genes or other unrelated genes, it is conceivable that they may function as oncogenes or tumor suppressors or both. To date, the majority of these studies support the notion that pseudogenes impact tumorigenesis through ceRNA mechanism.

As the first pseudogene identified to be able to regulate its parent gene *PTEN* through ceRNA mechanism, *PTEN*P1 is a tumor suppressor. *PTEN* is a well-known tumor suppressor and it serves a negative regulator for AKT. The phosphatase activity of *PTEN* is able to dephosphorylate tyrosine-, serine- and threonine-phosphorylated proteins. At the same time, *PTEN* also acts as a lipid phosphatase, dephosphorylating phosphoinositides and thus antagonizing the PI3K-AKT/PKB signaling pathway. Loss or inactivation of *PTEN*, which occurs in many tumor types, leads to increased RTK/PI3K/AKT signaling, thus, serving as a tumorigenesis driving force.

Regulation of *PTEN* is complex, including epigenetic, transcriptional, and post-transcriptional mechanisms as well as post-translational modification such as phosphorylation, acetylation and oxidization [56]. In particular, several microRNAs such as miR-21 have been shown to regulate *PTEN* by directly targeting the 3′-UTR of *PTEN* [57]. Since both *PTEN*P1 and *PTEN* share multiple MREs, these microRNAs can simultaneously regulate *PTEN*P1 and *PTEN* [18]. However, cellular content may alter this type of regulation. A recent study suggests that the *PTEN*P1-mediated regulation of *PTEN* is dependent on ER status in breast cancer [58]. For example, *PTEN*P1 upregulation decreases *PTEN* gene expression in the ER-positive breast cancer cells. Furthermore, *PTEN*P1 transduction significantly decreases ERα mRNA and protein levels in MCF7 xenografts with a concomitant increase in miR-26a, a microRNA known to target ERα. In contrast, in the ER-negative breast cancer cells, upregulation of *PTEN*P1 increases *PTEN* gene expression with no influence on miR-26a or ERα expression, but is able to reduce tumor metastasis in a xenograft model [58]. This divergent effect of *PTEN*P1 may reflect the complexity of pseudogene-mediated gene regulation, highlighting the importance of cellular content.

Several other studies support the notion that *PTEN*P1 is a tumor suppressor in variety of cancers, including esophageal squamous cell carcinoma [59], oral squamous cell carcinoma [60] head and neck squamous cell carcinoma [61] and melanoma [62]. *PTEN*P1 is also downregulated in clear-cell renal cell carcinoma tissues, and its expression is positively correlated with *PTEN* expression. Of interest, *PTEN*P1 can sensitize clear-cell renal cell carcinoma cells to cisplatin and gemcitabine treatments [63].

The pseudogene Foxo3P also functions as a tumor suppressor in breast cancer [64]. It is well known that the forkhead family of transcription factors plays important roles in regulating the expression of genes involved in cell growth, proliferation, cell apoptosis and survival, and development. For example, Foxo3 has been implicated in autophagy [65,66]. A recent study suggests that pseudogene Foxo3P, along with Foxo3 circular RNA, can regulate the parent gene Foxo3. Both Foxo3 and Foxo3P share a number of microRNA biding sites. Ectopic expression of the Foxo3P, Foxo3 circular RNA and Foxo3 mRNA suppress tumor growth, and cancer cell proliferation and survival [64].

BRAF plays in important role in cell signaling involving MAP kinase, leading to cell growth and proliferation. BRAF is often mutated in various types of cancer. An early study indicated that a BRAF pseudogene is mapped near the active gene [67]. In thyroid cancer, BRAF pseudogene expression is negatively associated with BRAF mutation because the pseudogene transcripts are more frequently detected in tumors without BRAF mutation than those with BRAF mutation [68]. Evidently, like the parent gene BRAF, BRAF pseudogene plays an oncogenic role by activating the MAP kinase signaling pathway, leading to the formation of tumors in nude mice. However, a recent study suggests that BRAF pseudogene mRNA levels are positively correlated with BRAF mRNA levels [69], which could be through ceRNA mechanism.

The oncogenic role of BRAF pseudogene in cancer comes from a study with animal models. For example, transgenic mice with the full-length murine BRAF pseudogene BRAF-rs1 or its pseudo “CDS” or “3′-UTR” develop an aggressive malignancy resembling human diffuse large B cell lymphoma [20]. This BRAF pseudogene-induced tumorigenesis is dependent on Dicer1, a key enzyme for microRNA processing [70]. Furthermore, a group of microRNAs such as miR-134, miR-543, and miR-653 can significantly suppress the activity of BRAF-rs1 and BRAF luciferase reporters. Finally, there are transcriptional or genomic aberrations of human BRAF pseudogene (BRAFP1) frequently in multiple human cancers, including B cell lymphomas, suggesting the clinical significance of BRAF pseudogene [20].

DUXAP10 is upregulated in various types of cancer [71,72,73] and its expression is positively associated with cancer progression and/or metastasis. For instance, the level of DUXAP10 is significantly increased in pancreatic cancer patients with an advanced TNM stage and positive lymph node metastasis. Mechanistically, DUXAP10 regulates cell proliferation through regulation of cell cycle, which involves the interaction with RNA-binding protein EZH2 and LSD1 [72]. Thus, DUXAP10 is an oncogenic pseudogene.

Iron homeostasis is critical to maintenance of normal cellular functions. Dysregulation of iron metabolism can promote cancer growth and survival. In this regard, ferritin heavy chain 1 (FTH1) is a key subunit of the ferritin that stores iron in its non-toxic ferric form, and it plays a critical role in the maintenance of iron homeostasis in cells to prevent harmful effects caused by iron overload. A number of FTH1 pseudogenes have been shown to regulate FTH1 through ceRNA mechanism, involving multiple microRNAs and FTH1 pseudogenes, contributing to prostate cancer development and progression [74]. FTH1P3 also plays an oncogenic role in uveal melanoma cells. Overexpression of FTH1P3 promotes uveal melanoma cell proliferation and migration, which involves miR-224-5p and its direct target genes Rac1 and Fizzled 5 [75].

CYP4Z1 is a member of the cytochrome P450 superfamily of enzymes that catalyze many reactions involved in drug metabolism and synthesis of cholesterol, steroids and other lipids. CYP4Z1 is overexpressed in various types of cancer. However, how CYP4Z1 is regulated in cancer is not well understood. Several studies suggest that the pseudogene CYP4Z2P is a positive regulator of CYP4Z1 through the interaction with multiple microRNAs. This ceRNA network regulates tumor angiogenesis, and tamoxifen resistance [76,77] and even expression of hTERT [78].

There are still more examples of pseudogenes as oncogenes, including CTNNAP1 and PDIA3P1, DUXAP8 and PHBP1 [79,80,81,82]. Overall, it appears that more oncogenic pseudogenes have been identified than tumor suppressive pseudogene probably because most of these pseudogenes function as positive regulators of their parent genes that are oncogenic.

To further demonstrate the clinical significance of pseudogenes, we examined alterations of pseudogene from TCGA dataset (c-BioPortal) [83,84]. Overall, copy number alteration (CNA) for a number of pseudogenes occurs broadly in cancer. For example, we interrogated 13,000 annotated pseudogenes from HGNC against five major solid tumors (breast, colon, lung, prostate and melanoma), and found that a roughly 10% of these 13,000 pseudogenes revealed alterations of either CNA and/or RNA expression. Thus, we selected four pseudogenes (NACAP1, ZNF252P, FAM86B3P and RPL23AP53) for further analysis because they are top candidates that showed dysregulation in multiple cancer types. A major alteration for NACAP1 and ZNF252P was amplification in all 5 cancer types. In breast cancer the amplification rate for NACAP1 and ZNF252P was 17% and 15%, respectively. In four other cancers, the amplification of these two pseudogenes were roughly 5%. In contrast, a major alteration for FAM86B3P and RPL23AP53 was deletion. Over 8% alteration rate was detected for these two pseudogenes in prostate cancer. In colon, breast and lung cancer, both revealed about 6% of deletion rate. In melanoma, there was about 2% of deletion rate. At the same time, we also noticed about 1~2% of amplification. Overall for those four pseudogenes, the mutation rates were not detectable or very low.

**Figure 3:**
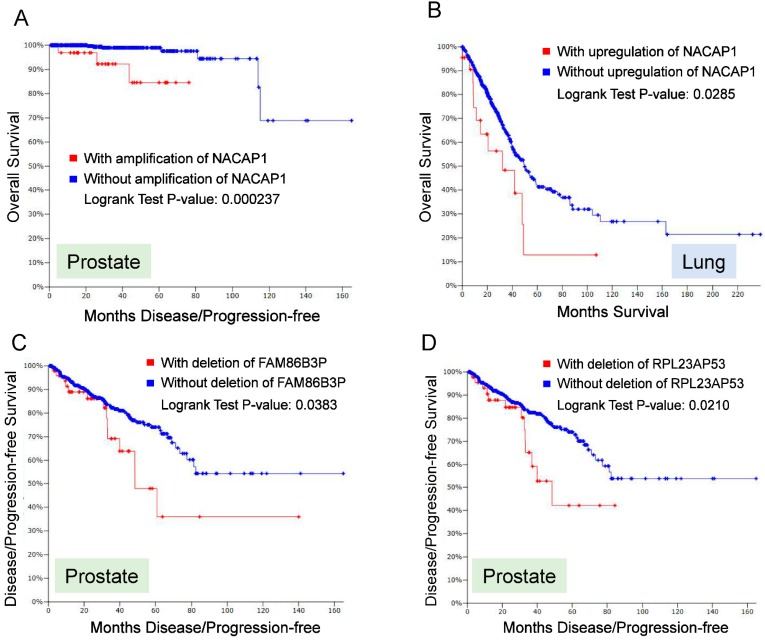
Alterations of pseudogenes are associated with overall survival or disease free survival.

Of interest, these alterations (amplification or deletion) in some pseudogenes was associated with poor prognosis (Figure 3). Among those four pseudogenes, amplification of NACAP1 was associated with overall survival in prostate cancer (Figure 3A); upregulation of NACAP1 was associated with overall survival in lung cancer (Figure 3B). In addition, deletion of FAM86B3P and RPL23AP53 was associated with disease-free survival in prostate cancer (Figure 3C,D). These results suggest that alterations of pseudogenes occur broadly in various types of cancer, and they may serve as diagnostic or prognostic markers, further highlighting the clinical significance of pseudogenes. 

## 5. Challenges for Pseudogene Research

To date, there is substantial evidence that pseudogenes play an important role in cancer. However, their function and underlying mechanisms largely remain to be determined yet. Available evidence points ceRNA mechanism for pseudogene-mediated gene regulation as a major mechanism probably because this type of gene regulation is relatively easy to study. However, as discussed above, ceRNA mechanism is not the only one and other mechanisms could be also important for pseudogene-mediated gene regulation. As a member of lncRNA family, pseudogenes may also play similar roles in gene regulation as what have been demonstrated for lncRNAs. For example, pseudogenes may also function as a scaffold to bring different components (e.g., DNA, RNA and protein) together to form a functional complex.

A great challenge for pseudogene studies stems from the fact that there is a high sequence homology between parent genes and their pseudogenes with an exception for unitary pseudogenes that lack their parent genes. This sequence homology makes it very difficult to specifically detect and target pseudogenes. Thus, the lack of pseudogene-specific primers or probes makes the detection of pseudogenes by conventional array, RT-PCR or in situ hybridization unreliable. Furthermore, due to lack of protein-coding capacity, we are not able to detect their expression using immunostaining methods such immunohistochemistry or immunofluorescent microscopy or western blot. Even for those pseudogenes that do make proteins, there is often lack of suitable antibodies that can be used in immunodetection methods. In addition, functional studies of pseudogenes often involve the manipulation of the target genes such as knockout or knockdown. However, due to high sequence homology to parent gene, it is often difficult to perform this type of experiments. To tackle this challenge, we may target intron regions that tend to be less conserved than the exon regions by CRISPR/Cas9 dual gRNA approach [85]. Lastly, pseudogene expression discovery by RNA-seq analysis often encounters with the difficulty to uniquely identify reads mapped to pseudogene regions. In this case, long range of reads may help to improve the accuracy of sequence alignment.

## 6. Concluding Remarks and Future Directions

Like other types of lncRNA, pseudogenes are important part of gene regulatory network, ultimately impacting tumorigenesis. Dysregulation of pseudogenes in clinical specimens and studies with cell culture and animal models all support the role of pseudogenes in cancer. It is evident that pseudogenes can function as oncogenes or tumor suppressors. A major function mechanism is that pseudogenes can serve as microRNA decoys to compete microRNAs that may target parent genes. Therefore, pseudogenes may serve as potential diagnostic or prognostic markers.

Pseudogenes were discovered related to their parent protein-coding genes, because they have lost their initial function, i.e., coding capacity. By analogy, we expect that lncRNAs may also have their “pseudogenes”. A challenge to define this type of pseudogenes is evident because the function for vast majority of lncRNAs is still unknown. Nevertheless, a pseudogene can be derived from the lncRNA such that the pseudogene may be involved in regulation of this lncRNA. Therefore, the repertoires of pseudogenes can be further increased and their function could be even more complex.

With regard to mechanisms of pseudogenes, in addition to what we have discussed above, there may exist other types of gene regulation, such as RNA methylation. It is known that RNA methylation (m^6^A) can change structure of mRNAs that can lead to different RNA-protein interactions such that RNA methylation can affect RNA stability and splicing [86,87]. For example, MALAT1 methylation can enhance its interaction with splicing factors such as heterogeneous nuclear ribonucleoprotein C (hnRNP C) [86], lending to different splicing patterns. Pseudogenes are a special class of lncRNAs. Thus, pseudogenes may possess all functional capability as other types of lncRNAs. Therefore, pseudogene research is a wide open area to be explored.

In summary, pseudogene research is still at a very early stage and there is a lot to be learned. Future work should focus on functional characterization of pseudogenes. In this regard, functional screening from RNAi libraries or CRISPR-based libraries at the genome wide scale would provide a comprehensive view of pseudogenes (expression and function). Further characterization of these pseudogenes will help us to better understand pseudogenes and their role in cancer. As a result, like lncRNAs, pseudogenes may serve as cancer biomarkers or therapeutic targets.

## References

[1] Esteller M. (2011). Non-coding RNAs in human disease. Nat. Rev. Genet..

[2] Wilusz J.E. (2016). Long noncoding RNAs: Re-writing dogmas of RNA processing and stability. Biochim. Biophys. Acta.

[3] Wang Y., Chen L., Chen B., Li X., Kang J., Fan K., Hu Y., Xu J., Yi L., Yang J. (2013). Mammalian ncRNA-disease repository: A global view of ncRNA-mediated disease network. Cell Death Dis..

[4] Fatica A., Bozzoni I. (2014). Long non-coding RNAs: New players in cell differentiation and development. Nat. Rev. Genet..

[5] Garzon R., Calin G.A., Croce C.M. (2009). MicroRNAs in cancer. Annu. Rev. Med..

[6] Prensner J.R., Chinnaiyan A.M. (2011). The emergence of lncRNAs in cancer biology. Cancer Discov..

[7] Wahlestedt C. (2013). Targeting long non-coding RNA to therapeutically upregulate gene expression. Nat. Rev. Drug Discov..

[8] Zhao Y., Li H., Fang S., Kang Y., Wu W., Hao Y., Li Z., Bu D., Sun N., Zhang M.Q. (2016). Noncode 2016: An informative and valuable data source of long non-coding RNAs. Nucleic Acids Res..

[9] Mestdagh P., Fredlund E., Pattyn F., Rihani A., van Maerken T., Vermeulen J., Kumps C., Menten B., De Preter K., Schramm A. (2010). An integrative genomics screen uncovers ncRNA T-UCR functions in neuroblastoma tumours. Oncogene.

[10] Marques A.C., Ponting C.P. (2014). Intergenic lncRNAs and the evolution of gene expression. Curr. Opin. Genet. Dev..

[11] Ulitsky I., Bartel D.P. (2013). LincRNAs: Genomics, evolution, and mechanisms. Cell.

[12] Mattick J.S., Rinn J.L. (2015). Discovery and annotation of long noncoding RNAs. Nat. Struct. Mol. Biol..

[13] Katayama S., Tomaru Y., Kasukawa T., Waki K., Nakanishi M., Nakamura M., Nishida H., Yap C.C., Suzuki M., Kawai J. (2005). Antisense transcription in the mammalian transcriptome. Science.

[14] Khorkova O., Myers A.J., Hsiao J., Wahlestedt C. (2014). Natural antisense transcripts. Hum. Mol. Genet..

[15] Natoli G., Andrau J.C. (2012). Noncoding transcription at enhancers: General principles and functional models. Annu. Rev. Genet..

[16] Carninci P., Kasukawa T., Katayama S., Gough J., Frith M.C., Maeda N., Oyama R., Ravasi T., Lenhard B., Wells C. (2005). The transcriptional landscape of the mammalian genome. Science.

[17] Balakirev E.S., Ayala F.J. (2003). Pseudogenes: Are they “junk” or functional DNA?. Annu. Rev. Genet..

[18] Poliseno L., Salmena L., Zhang J., Carver B., Haveman W.J., Pandolfi P.P. (2010). A coding-independent function of gene and pseudogene mRNAs regulates tumour biology. Nature.

[19] Lujambio A., Lowe S.W. (2012). The microcosmos of cancer. Nature.

[20] Karreth F.A., Reschke M., Ruocco A., Ng C., Chapuy B., Leopold V., Sjoberg M., Keane T.M., Verma A., Ala U. (2015). The BRAF pseudogene functions as a competitive endogenous RNA and induces lymphoma in vivo. Cell.

[21] Poliseno L. (2012). Pseudogenes: Newly discovered players in human cancer. Sci. Signal..

[22] Poliseno L., Marranci A., Pandolfi P.P. (2015). Pseudogenes in human cancer. Front. Med..

[23] Ng S.Y., Gunning P., Eddy R., Ponte P., Leavitt J., Shows T., Kedes L. (1985). Evolution of the functional human beta-actin gene and its multi-pseudogene family: Conservation of noncoding regions and chromosomal dispersion of pseudogenes. Mol. Cell. Biol..

[24] McDonell L., Drouin G. (2012). The abundance of processed pseudogenes derived from glycolytic genes is correlated with their expression level. Genome.

[25] Robicheau B.M., Susko E., Harrigan A.M., Snyder M. (2017). Ribosomal RNA genes contribute to the formation of pseudogenes and junk DNA in the human genome. Genome Biol. Evolut..

[26] Guo X., Lin M., Rockowitz S., Lachman H.M., Zheng D. (2014). Characterization of human pseudogene-derived non-coding RNAs for functional potential. PLoS ONE.

[27] Bossi L., Figueroa-Bossi N. (2016). Competing endogenous RNAs: A target-centric view of small RNA regulation in bacteria. Nat. Rev. Microbiol..

[28] Thomson D.W., Dinger M.E. (2016). Endogenous microRNA sponges: Evidence and controversy. Nat. Rev. Genet..

[29] Tay Y., Rinn J., Pandolfi P.P. (2014). The multilayered complexity of ceRNA crosstalk and competition. Nature.

[30] Zhang Z., Zhu Z., Watabe K., Zhang X., Bai C., Xu M., Wu F., Mo Y.Y. (2013). Negative regulation of lncRNA gas5 by miR-21. Cell Death Differ..

[31] Liu Q., Huang J., Zhou N., Zhang Z., Zhang A., Lu Z., Wu F., Mo Y.Y. (2013). LncRNA loc285194 is a p53-regulated tumor suppressor. Nucleic Acids Res..

[32] Rodriguez A., Griffiths-Jones S., Ashurst J.L., Bradley A. (2004). Identification of mammalian microRNA host genes and transcription units. Genome Res..

[33] Devor E.J. (2006). Primate microRNAs miR-220 and miR-492 lie within processed pseudogenes. J. Hered..

[34] DeMaria C.T., Brewer G. (1996). AUF1 binding affinity to A+U-rich elements correlates with rapid mRNA degradation. J. Biol. Chem..

[35] Gratacos F.M., Brewer G. (2010). The role of AUF1 in regulated mRNA decay. Wiley Interdiscip. Rev. RNA.

[36] Bernasconi N.L., Wormhoudt T.A., Laird-Offringa I.A. (2000). Post-transcriptional deregulation of *Myc* genes in lung cancer cell lines. Am. J. Respir. Cell Mol. Biol..

[37] Huang J., Zhang A., Ho T.T., Zhang Z., Zhou N., Ding X., Zhang X., Xu M., Mo Y.Y. (2015). Linc-ROR promotes *c-Myc* expression through hnRNP I and AUF1. Nucleic Acids Res..

[38] Haddadi N., Lin Y., Travis G., Simpson A.M., McGowan E.M., Nassif N.T. (2018). *PTEN*/*PTEN*p1: ‘Regulating the regulator of RTK-dependent Pi3K/AKT signalling’, new targets for cancer therapy. Mol. Cancer.

[39] Johnsson P., Ackley A., Vidarsdottir L., Lui W.O., Corcoran M., Grander D., Morris K.V. (2013). A pseudogene long-noncoding-RNA network regulates *PTEN* transcription and translation in human cells. Nat. Struct. Mol. Biol..

[40] Lister N., Shevchenko G., Walshe J.L., Groen J., Johnsson P., Vidarsdottir L., Grander D., Ataide S.F., Morris K.V. (2017). The molecular dynamics of long noncoding RNA control of transcription in *PTEN* and its pseudogene. Proc. Natl. Acad. Sci. USA.

[41] Wezel F., Pearson J., Kirkwood L.A., Southgate J. (2013). Differential expression of OCT4 variants and pseudogenes in normal urothelium and urothelial cancer. Am. J. Pathol..

[42] Suo G., Han J., Wang X., Zhang J., Zhao Y., Dai J. (2005). OCT4 pseudogenes are transcribed in cancers. Biochem. Biophys. Res. Commun..

[43] Hayashi H., Arao T., Togashi Y., Kato H., Fujita Y., De Velasco M.A., Kimura H., Matsumoto K., Tanaka K., Okamoto I. (2015). The OCT4 pseudogene POU5F1B is amplified and promotes an aggressive phenotype in gastric cancer. Oncogene.

[44] Breyer J.P., Dorset D.C., Clark T.A., Bradley K.M., Wahlfors T.A., McReynolds K.M., Maynard W.H., Chang S.S., Cookson M.S., Smith J.A. (2014). An expressed retrogene of the master embryonic stem cell gene POU5F1 is associated with prostate cancer susceptibility. Am. J. Hum. Genet..

[45] Sumter T.F., Xian L., Huso T., Koo M., Chang Y.T., Almasri T.N., Chia L., Inglis C., Reid D., Resar L.M. (2016). The high mobility group a1 (HMGA1) transcriptome in cancer and development. Curr. Mol. Med..

[46] De Martino M., Forzati F., Arra C., Fusco A., Esposito F. (2016). HMGA1-pseudogenes and cancer. Oncotarget.

[47] Brunetti A., Manfioletti G., Chiefari E., Goldfine I.D., Foti D. (2001). Transcriptional regulation of human insulin receptor gene by the high-mobility group protein HMGI(Y). FASEB J..

[48] Foti D., Iuliano R., Chiefari E., Brunetti A. (2003). A nucleoprotein complex containing SP1, C/EBP beta, and HMGI-y controls human insulin receptor gene transcription. Mol. Cell. Biol..

[49] Chiefari E., Iiritano S., Paonessa F., le Pera I., Arcidiacono B., Filocamo M., Foti D., Liebhaber S.A., Brunetti A. (2010). Pseudogene-mediated posttranscriptional silencing of *HMGA1* can result in insulin resistance and type 2 diabetes. Nat. Commun..

[50] Czyzyk-Krzeska M.F., Bendixen A.C. (1999). Identification of the poly(c) binding protein in the complex associated with the 3′ untranslated region of erythropoietin messenger RNA. Blood.

[51] Han Y.J., Ma S.F., Yourek G., Park Y.D., Garcia J.G. (2011). A transcribed pseudogene of MYLK promotes cell proliferation. FASEB J..

[52] Tam O.H., Aravin A.A., Stein P., Girard A., Murchison E.P., Cheloufi S., Hodges E., Anger M., Sachidanandam R., Schultz R.M. (2008). Pseudogene-derived small interfering RNAs regulate gene expression in mouse oocytes. Nature.

[53] Chan W.L., Yuo C.Y., Yang W.K., Hung S.Y., Chang Y.S., Chiu C.C., Yeh K.T., Huang H.D., Chang J.G. (2013). Transcribed pseudogene *PSIPPM1K* generates endogenous siRNA to suppress oncogenic cell growth in hepatocellular carcinoma. Nucleic Acids Res..

[54] Wen Y.Z., Zheng L.L., Liao J.Y., Wang M.H., Wei Y., Guo X.M., Qu L.H., Ayala F.J., Lun Z.R. (2011). Pseudogene-derived small interference RNAs regulate gene expression in African trypanosoma brucei. Proc. Natl. Acad. Sci. USA.

[55] Korneev S.A., Park J.H., O’Shea M. (1999). Neuronal expression of neural nitric oxide synthase (NNOS) protein is suppressed by an antisense RNA transcribed from an NOS pseudogene. J. Neurosci..

[56] Bermudez Brito M., Goulielmaki E., Papakonstanti E.A. (2015). Focus on *PTEN* regulation. Front. Oncol..

[57] Meng F., Henson R., Wehbe-Janek H., Ghoshal K., Jacob S.T., Patel T. (2007). MicroRNA-21 regulates expression of the *PTEN* tumor suppressor gene in human hepatocellular cancer. Gastroenterology.

[58] Yndestad S., Austreid E., Skaftnesmo K.O., Lonning P.E., Eikesdal H.P. (2018). Divergent activity of the pseudogene *PTEN*p1 in ER-positive and negative breast cancer. Mol. Cancer Res..

[59] Gong T., Zheng S., Huang S., Fu S., Zhang X., Pan S., Yang T., Sun Y., Wang Y., Hui B. (2017). *PTEN*p1 inhibits the growth of esophageal squamous cell carcinoma by regulating SOCS6 expression and correlates with disease prognosis. Mol. Carcinog..

[60] Gao L., Ren W., Zhang L., Li S., Kong X., Zhang H., Dong J., Cai G., Jin C., Zheng D. (2017). *PTEN*p1, a natural sponge of miR-21, mediates *PTEN* expression to inhibit the proliferation of oral squamous cell carcinoma. Mol. Carcinog..

[61] Liu J., Xing Y., Xu L., Chen W., Cao W., Zhang C. (2017). Decreased expression of pseudogene *PTEN*p1 promotes malignant behaviours and is associated with the poor survival of patients with HNSCC. Sci. Rep..

[62] Poliseno L., Haimovic A., Christos P.J., Vega Y.S.D.M.E.C., Shapiro R., Pavlick A., Berman R.S., Darvishian F., Osman I. (2011). Deletion of *PTENp1* pseudogene in human melanoma. J. Investig. Dermatol..

[63] Yu G., Yao W., Gumireddy K., Li A., Wang J., Xiao W., Chen K., Xiao H., Li H., Tang K. (2014). Pseudogene *PTENp1* functions as a competing endogenous RNA to suppress clear-cell renal cell carcinoma progression. Mol. Cancer Ther..

[64] Yang W., Du W.W., Li X., Yee A.J., Yang B.B. (2016). FOXO3 activity promoted by non-coding effects of circular RNA and FOXO3 pseudogene in the inhibition of tumor growth and angiogenesis. Oncogene.

[65] Gomez-Puerto M.C., Verhagen L.P., Braat A.K., Lam E.W., Coffer P.J., Lorenowicz M.J. (2016). Activation of autophagy by FOXO3 regulates redox homeostasis during osteogenic differentiation. Autophagy.

[66] Fitzwalter B.E., Towers C.G., Sullivan K.D., Andrysik Z., Hoh M., Ludwig M., O’Prey J., Ryan K.M., Espinosa J.M., Morgan M.J. (2018). Autophagy inhibition mediates apoptosis sensitization in cancer therapy by relieving FOXO3a turnover. Dev. Cell.

[67] Sithanandam G., Druck T., Cannizzaro L.A., Leuzzi G., Huebner K., Rapp U.R. (1992). B-Raf and a B-Raf pseudogene are located on 7q in man. Oncogene.

[68] Zou M., Baitei E.Y., Alzahrani A.S., Al-Mohanna F., Farid N.R., Meyer B., Shi Y. (2009). Oncogenic activation of map kinase by BRAF pseudogene in thyroid tumors. Neoplasia.

[69] Lin J.D., Fu S.S., Chen J.Y., Lee C.H., Chau W.K., Cheng C.W., Wang Y.H., Lin Y.F., Fang W.F., Tang K.T. (2016). Clinical manifestations and gene expression in patients with conventional papillary thyroid carcinoma carrying the *BRAF^V600E^* mutation and *BRAF* pseudogene. Thyrod.

[70] Foulkes W.D., Priest J.R., Duchaine T.F. (2014). Dicer1: Mutations, microRNAs and mechanisms. Nat. Rev. Cancer.

[71] Lian Y., Xu Y., Xiao C., Xia R., Gong H., Yang P., Chen T., Wu D., Cai Z., Zhang J. (2017). The pseudogene derived from long non-coding RNA DUXAP10 promotes colorectal cancer cell growth through epigenetically silencing of p21 and *PTEN*. Sci. Rep..

[72] Lian Y., Xiao C., Yan C., Chen D., Huang Q., Fan Y., Li Z., Xu H. (2018). Knockdown of pseudogene derived from lncRNA DUXAP10 inhibits cell proliferation, migration, invasion, and promotes apoptosis in pancreatic cancer. J. Cell. Biochem..

[73] Xu Y., Yu X., Wei C., Nie F., Huang M., Sun M. (2018). Over-expression of oncigenic pesudogene DUXAP10 promotes cell proliferation and invasion by regulating LATS1 and beta-catenin in gastric cancer. J. Exp. Clin. Cancer Res..

[74] Chan J.J., Kwok Z.H., Chew X.H., Zhang B., Liu C., Soong T.W., Yang H., Tay Y. (2017). A fth1 gene:Pseudogene:MicroRNA network regulates tumorigenesis in prostate cancer. Nucleic Acids Res..

[75] Zheng X., Tang H., Zhao X., Sun Y., Jiang Y., Liu Y. (2017). Long non-coding RNA FTH1P3 facilitates uveal melanoma cell growth and invasion through miR-224-5p. PLoS ONE.

[76] Zheng L., Li X., Gu Y., Lv X., Xi T. (2015). The 3′UTR of the pseudogene *CYP4Z2P* promotes tumor angiogenesis in breast cancer by acting as a ce*RNA* for *CYP4Z1*. Breast Cancer Res. Treat..

[77] Zheng L., Li X., Meng X., Chou J., Hu J., Zhang F., Zhang Z., Xing Y., Liu Y., Xi T. (2016). Competing endogenous RNA networks of *CYP4Z1* and pseudogene *CYP4Z2P* confer tamoxifen resistance in breast cancer. Mol. Cell. Endocrinol..

[78] Li C., Zheng L., Xin Y., Tan Z., Zhang Y., Meng X., Wang Z., Xi T. (2017). The competing endogenous RNA network of *CYP4Z1* and pseudogene *CYP4Z2P* exerts an anti-apoptotic function in breast cancer. FEBS Lett..

[79] Chen X., Zhu H., Wu X., Xie X., Huang G., Xu X., Li S., Xing C. (2016). Downregulated pseudogene *CTNNAP*1 promote tumor growth in human cancer by downregulating its cognate gene ctnna1 expression. Oncotarget.

[80] Kong Y., Zhang L., Huang Y., He T., Zhao X., Zhou X., Zhou D., Yan Y., Zhou J., Xie H. (2017). Pseudogene *PDIA3P1* promotes cell proliferation, migration and invasion, and suppresses apoptosis in hepatocellular carcinoma by regulating the p53 pathway. Cancer Lett..

[81] Feng F., Qiu B., Zang R., Song P., Gao S. (2017). Pseudogene *PHBP1* promotes esophageal squamous cell carcinoma proliferation by increasing its cognate gene *PHB* expression. Oncotarget.

[82] Sun M., Nie F.Q., Zang C., Wang Y., Hou J., Wei C., Li W., He X., Lu K.H. (2017). The pseudogene *DUXAP*8 promotes non-small-cell lung cancer cell proliferation and invasion by epigenetically silencing *EGR1* and *RHOB*. Mol. Ther..

[83] Gao J., Aksoy B.A., Dogrusoz U., Dresdner G., Gross B., Sumer S.O., Sun Y., Jacobsen A., Sinha R., Larsson E. (2013). Integrative analysis of complex cancer genomics and clinical profiles using the cbioportal. Sci. Signal..

[84] Cerami E., Gao J., Dogrusoz U., Gross B.E., Sumer S.O., Aksoy B.A., Jacobsen A., Byrne C.J., Heuer M.L., Larsson E. (2012). The CBIO cancer genomics portal: An open platform for exploring multidimensional cancer genomics data. Cancer Discov..

[85] Ho T.T., Zhou N., Huang J., Koirala P., Xu M., Fung R., Wu F., Mo Y.Y. (2015). Targeting non-coding RNAs with the CRISPR/CAS9 system in human cell lines. Nucleic Acids Res..

[86] Liu N., Dai Q., Zheng G., He C., Parisien M., Pan T. (2015). *N*(6)-methyladenosine-dependent RNA structural switches regulate RNA-protein interactions. Nature.

[87] Wang X., Lu Z., Gomez A., Hon G.C., Yue Y., Han D., Fu Y., Parisien M., Dai Q., Jia G. (2014). *N*6-methyladenosine-dependent regulation of messenger RNA stability. Nature.

